# Health and Psychosocial Risks of Unengaged Residents in Low-Income Independent Living Communities

**DOI:** 10.1093/geroni/igaf122.4129

**Published:** 2025-12-31

**Authors:** Jeremy Johnson, Khalid Yusuf, Emily Campbell, Autumn Hart, Victor Ronis-Tobin

**Affiliations:** Xavier University, Cincinnati, Ohio, United States; University of Cincinnati, Cincinnati, Ohio, United States; Xavier University, Cincinnati, Ohio, United States; Xavier University, Cincinnati, Ohio, United States; Xavier University, Cincinnati, Ohio, United States

## Abstract

Social engagement is a critical determinant of health in older adults, yet many residents of independent living communities remain unengaged despite available opportunities. We report baseline data from a longitudinal wellness program study of residents in low-income independent communities in Southwest Ohio. Assessments measured general health, quality of life, and psychosocial function. Participants (N = 119) were older adults (M = 73.5, SD = 8.6), primarily female (83.2%), African American (60.5%), and with annual incomes below $25,000 (86%). Quartile splits of the Social Engagement and Activities Questionnaire (SEAQ) were used to define engagement, with most participants (69.9%) classified as engaged. Compared to engaged peers, unengaged residents (31.1%) scored significantly lower on domains of physical functioning (M = –12.27, p = .020), vitality (–9.86, p = .016), and emotional well-being (–10.26, p = .013), on the SF–36 quality of life instrument, and had higher fall risk scores (M = 1.37, p = .035) on a fall risk assessment. Other SF-36 domains trended in the expected direction but were nonsignificant. Unengaged residents also showed nonsignificant trends toward greater loneliness scores (UCLA-3 Item) and healthcare utilization (annual hospital visits). Examining social isolations using the Lubben Social Network Scale (LSNS-6), unengaged residents were nearly three times more likely to report social isolation than engaged residents (32.4% vs. 12.2%; OR = 3.46, 95% CI [1.33–8.98]). Findings highlight the need to improve engagement in wellness programming of older low-income adults living independently.

